# Developing a Feasible and Credible Method for Analyzing Healthcare Documents as Written Data

**DOI:** 10.1177/23333936221108706

**Published:** 2022-07-07

**Authors:** Tanja Moilanen, Mari Sivonen, Kirsi Hipp, Hanna Kallio, Oili Papinaho, Minna Stolt, Riitta Turjamaa, Arja Häggman-Laitila, Mari Kangasniemi

**Affiliations:** 1 University of Turku, Finland; 2 Karelia University of Applied Sciences, Joensuu, Finland; 3 Häme University of Applied Sciences, Finland; 4 Oulu University Hospital, Finland; 5 Savonia University of Applied Sciences, Kuopio, Finland; 6 University of Eastern Finland, Kuopio, Finland; 7 Department of Social Services and Health Care, Helsinki, Finland

**Keywords:** credibility, document analysis, feasibility, methodology, systematic methodological review, written data

## Abstract

Healthcare provides a rich, and constantly increasing, number of written documents, which are underutilized in research data for health and nursing sciences, but previous literature has only provided limited guidance on the process of document analysis. The aim of this paper is to provide a methodological framework for analyzing health care documents as written data, based on a systematic methodological review and the research team’s experience of the method. Based on the results, the methods consist of seven phases: (i) identify the purpose, (ii) determine the document selection strategy, (iii) select or design an extraction matrix, (iv) carry out pilot testing, (v) collect and analyze the data, (vi) consider the credibility, and (vii) ethics of the study. The framework that has been developed can be used to carry out document analysis studies that are both feasible and credible.

## Introduction

Document analysis is a topical method used in health and nursing sciences. Written, audio, and visual healthcare documents are constantly being produced (Bowen, 2009; Coffey, 2014; Gibson & Brown, 2011) and the number of documents is increasing (Olivares Bøgeskov & Grimshaw-Aagaard, 2019), because of wider healthcare regulations and the need to evaluate the effectiveness of care and services. Most of these healthcare documents are publicly available. The strength is that researchers have had no influence on their production, but the limitation is that data in healthcare documents have not been produced for research purposes (Bowen, 2009; Gross, 2018; Miller & Alvarado, 2005; Olson, 2012) However, healthcare documents can provide knowledge that cannot be obtained by other methods. Document analysis is also a topical research method, because of the increased production of digital healthcare documents and the use of artificial intelligence to carry out data mining in health sciences (Mehta & Pandit, 2018; Sundermann et al., 2019). Despite the topicality of the document analysis method, previous methodological literature have only proposed fragmented guidance on this research method (Bowen, 2009; Miller & Alvarado, 2005).

### Background

Document analysis refers to a systematic process of reviewing and analyzing documents (Kaae & Traulsen, 2015; Mercieca et al., 2019). It has been used as an independent method and has also been combined with other research methods (Bowen, 2009; Olson, 2012; Siegner et al., 2018). The advantage of document analysis is that it can produce new and trustworthy knowledge (Bowen, 2009; Gibson & Brown, 2011; Siegner et al., 2018) on study topics that cannot be empirically studied (Bowen, 2009; Siegner et al., 2018), but the disadvantage is that usually the documents requires pre-working and multiple research skills (Bowen, 2009).

This review regards healthcare documents as written data that have been produced, or used, to steer, organize, and implement care and services. For example, international and national steering documents aim to regulate and ensure the quality and availability of services (Ritter et al., 2018) and to support the management and organization of healthcare. On an organizational level, healthcare documents have been used to regulate and guide the implementation of practices that aim to ensure conformity and quality of services. When it comes to implementation, documents have been used to plan, record, and evaluate care (Olivares Bøgeskov & Grimshaw-Aagaard, 2019; Walker et al., 2018).

Healthcare documents can be official or unofficial (Coffey, 2014; Gibson & Brown, 2011). Most healthcare documents are official responses to legislative requirements or stakeholders’ rights. They can comprise patient records, national and organizational health plans, and annual reports, but also include complaints from clients and patients. One example of unofficial documents is instructions for care practices. In addition, the security level of healthcare documents varies. For example, public health plans and national care guidelines are publicly available, complaints or disciplinary decisions related how healthcare staff are managed are classified. Also, the structure of the document data may provide heterogeneity within, and between, the documents. For example, client complaints can be structured, but also include free, manual text.

## Rewiew

### Aims

The aim of this paper is to provide a methodological framework for analyzing health care documents as written data, based on a systematic methodological review and the research team’s experience of the method. The ultimate aim was to identify the different phases of the document analysis method and the feasibility and credibility of this research process.

### Design

We used systematic methodological review design by applying the theory review method (Campbell et al., 2014) with the Preferred Reporting Items for Systematic Reviews and Meta-Analyses checklist (Martín-Rodero et al., 2018; Moher et al., 2009) to identify previous methodological literature on document analysis. In addition, we used our experience of carrying out 14 studies using document analysis to examine the feasibility and credibility of the method. These document analyses employed qualitative and quantitative methods and used documents such as patient records or plans (Häggman-Laitila, 2003; Häggman-Laitila et al., 2010, 2019, 2020; Hipp et al., 2020, 2021; Puustinen et al., 2021; Toivonen et al., 2020; Turjamaa et al., 2015), healthcare steering documents (Kallio et al., 2018, 2020), clients’ complaints (Kangasniemi et al., 2022), and administrative healthcare decisions (Papinaho et al., 2021, 2022).

### Search Methods

**Figure 1.**
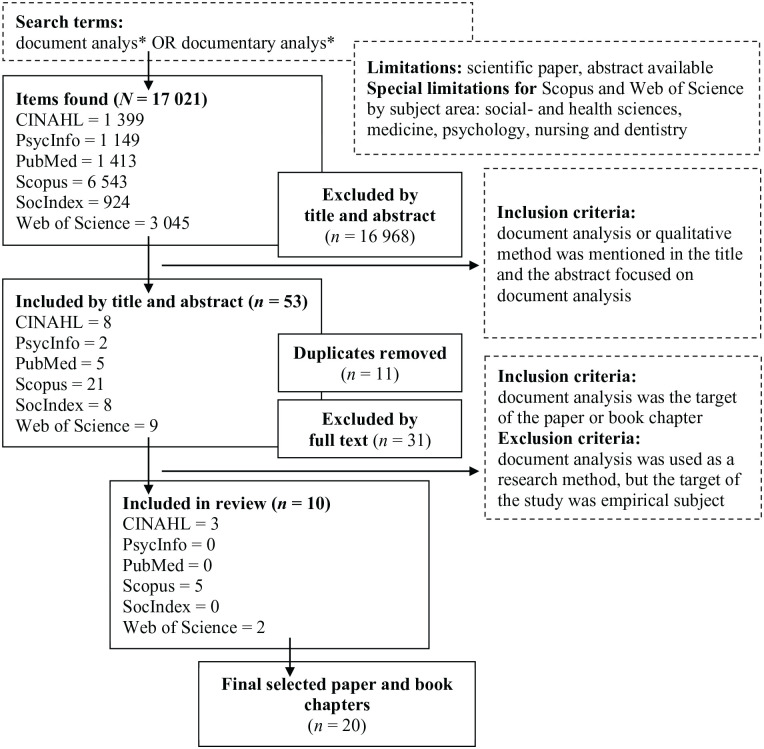
Flow chart of the literature searches and selection.

The literature searches were conducted (Campbell et al., 2014) using the CINAHL, PsycInfo, PubMed, Scopus, SocInde, and Web of Science databases (Figure 1). We determined the search terms by carrying out preliminary searches and consulting an information specialist and used the same search terms in the all databases. We limited the searches to scientific papers and book chapters that were published in the electronic databases from inception to May 2021 and had an abstract available. To make sure that our searches were comprehensive, we used general search terms as document analys* OR documentary analys* which were identified based on our preliminary searches. In addition, we also carried out manual searches of the reference lists of the selected papers. The electronic searches and the screening of reference lists were both limited to scientific papers and book chapters.

The database searches identified 17,021 publications. According to the inclusion and exclusion criteria, we selected 53 papers based on their title and abstract and 10 on their full text (Figure 1). A further 10 papers were identified by the manual searches of the reference lists, and this meant that the final analysis comprised 20 publications. The selection of the publications was independently conducted by three authors (TM, MS, and MK), who then worked together to finalize the list.

The publications were selected based on predetermined inclusion and exclusion criteria (Campbell et al., 2014). The inclusion criteria were that document analysis or corresponding methods were mentioned in the title and that abstract focused on document analysis. The inclusion criteria for the full texts were that document analysis was the target of the paper. Papers were excluded if they had used document analysis as a research method, but the study focused on an empirical subject. We did not limit what disciplines were covered by the publications.

### Search Outcomes

The 20 publications that we selected were published between 1981 and 2018 and comprised 12 scientific papers and 8 book chapters. As a concept, 11 used document analysis, 3 documentary analysis and documentary research, 2 the analysis of documentary realities or sources, and 1 documentary method. There were 18 literature-based discussions, 1 review, and 1 commentary. About 6 of the 11 papers came from the United Kingdom, 3 from the USA, and 2 from Australia and Canada. Seven of the eight book chapters did not include any information on the country or origin and the one that did come from the United Kingdom. About 5 of the 11 papers covered health science, 5 social science, and 1 marketing. Nine papers did not specify the discipline (Table 1).

**Table 1.** Description of the Selected Publications.

Author(s), year, country, type of publication	Aim of the publication	Method and data (*n* = references)	Field of study	Concept used
Appleton and Cowley (1997), UK, paper.	To describe documentary analysis as a tool for studying clinical guidelines	Description based on examples and previous literature (*n* = 42)	Health sciences: nursing	Documentary analysis
Atkinson and Coffey (2010), UK, book chapter.	To describe some of the methodological issues of documentary research	Examination based on previous literature (*n* = 21) and examples	ns	Analysis of documentary realities
Bowen (2009), USA, paper.	To examine documents as data sources and discuss document analysis	Literature-based methodological examination (*n* = 35)	ns	Document analysis
Caulley (1983), Australia, paper.	To introduce, and give an overview of, document analysis	Examination based on literature (*n* = 22) and experiences	ns	Document analysis
Coffey (2014), ns, book chapter.	To explore documents as research data and examine methodological aspects of document analysis	Examination based on previous literature (*n* = 29) and examples	Social Sciences	Documentary research
Finnegan (2011), ns, book chapter.	To examine, and describe, document analysis as a research method	Discussion based on literature (*n* = 5) and perceptions	Social sciences	Analysis of documentary sources
Gibson and Brown (2011), ns, book chapter.	To demonstrate the value of documentary sources as research data	Discussion of document analysis with examples of cases	ns	Documentary research
Gorichanaz and Latham (2016), USA, paper.	To propose a framework for document analysis from multiple perspectives	Literature based philosophical discussion (*n* = 52)	ns	Document analysis
Gross (2018), ns, paper.	To explore documents as research data	Examination based on literature (*n* = 6) and perceptions	ns	Document analysis
Hall and Rist (1999), USA, paper.	To examine document analysis, interviews, and observations used in market research	Examination based on case study and previous literature (*n* = 11)	Marketing	Document analysis
Kaae and Traulsen (2015), UK, book chapter.	To describe document analysis and ethnography and their validity, and reliability, in the field of pharmacy research	Examination based on previous literature (*n* = 25) and examples	ns	Documentary method
Miller and Alvarado (2005), Canada, paper.	To present an overview of incorporating documents as data sources into nursing research	Analysis of the nature of documents using previous literature (*n* = 23).	Nursing sciences	Document analysis
Murray and Sixsmith (2002), UK, paper.	To outline document analysis process on email posts and archives	Literature-based discussion (*n* = 37)	Health sciences: psychology	Document analysis
O’Connor (2011), ns, book chapter.	To describe documentary analysis as a method, using discourse and policy analysis	Examination based on previous literature (*n* = 28)	Health sciences: palliative care	Document analysis
Olson (2012), ns, book chapter.	To introduce document analysis	Examination based on research experiences	ns	Document analysis
Platt (1981), UK, paper.	To examine problems related to documentary research	Commentary based on literature (*n* = 33)	Social sciences	Documentary research
Rasmussen et al. (2012), Australia, paper.	To describe document analysis in the field of child and adolescents mental health nursing	Descriptive commentary using literature (*n* = 24)	Health sciences: nursing	Document analysis
Scott (2011), ns, book chapter.	To present an overview of document analysis	Description based on research experiences	ns	Document analysis
Siegner et al. (2018), Canada, paper.	To describe methodological practices of document analysis in the field of social research	Systematic review (*n* = 139), electronic and manual searches, other literature (*n* = 66)	Social sciences: forests	Document analysis
Sixsmith and Murray (2001), UK, paper.	To outline ethical considerations related to documentary analysis of web-based data	Discussion paper based on previous literature (*n* = 49) and examples.	Social sciences	Documentary analysis

*Note*. ns = not stated.

### Data Analysis and Synthesis

The selected publications were read several times to gain an overall understanding of them. The titles, aims, methods, and main results were then tabulated (Campbell et al., 2014). The content of the publications were analyzed using the constant comparison method (Boeije, 2002; Olson, 2012). First, all the expressions about the document analysis method were extracted from the data and these were a couple of words, sentences, or paragraphs. After that, we grouped expressions about the different phases of document analysis, and questions on rigor and ethics, based on their similarities and differences. During this phase, we incorporated our methodological experiences and findings based on the 14 scientific, peer-reviewed papers published by our team and used them to illustrate and elaborate different phases of the document analysis. We synthesized the data by naming the phases inductively. Because the wording and terminology used in expressions varied, we constantly compared the original text in the publications to preserve the original meaning of the expressions. After the process of document analysis was formulated, we illustrated the phases based our previous experiences of using the document analysis method.

## Results

**Figure 2.**
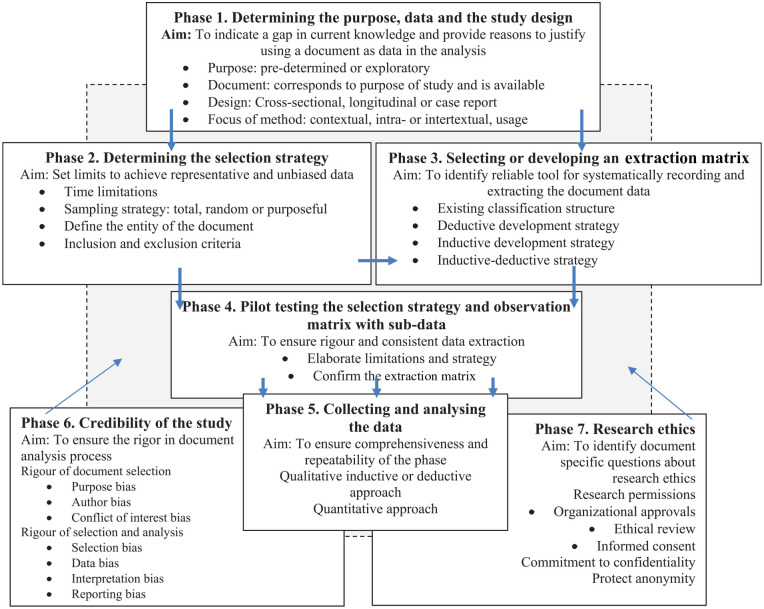
Phases of document analysis.

Our results indicated that the rigorous document analysis process consists of seven interlinked phases (Figure 2). Although the phases are presented separately, they can simultaneously overlap with each other and two phases were integrated with the other five.

### First Phase: Determining the Purpose, Data, and Study Design

The first phase of document analysis is to determine the *purpose* of the study and the data sources to be used by the study (Figure 2). The aim of this phase is to indicate any gaps in current knowledge and identify documents that can be used for research purposes. Document analysis aims to identify or clarify knowledge (Bowen, 2009; Gross, 2018; Olson, 2012; Siegner et al., 2018) by analyzing, synthetizing, or interpreting the study topic. In addition, the aim can be to describe or explain the meanings, patterns (Kaae & Traulsen, 2015), classifications, or processes of the study topic. Document analysis produces retrospective knowledge (Gibson & Brown, 2011; Gross, 2018; Olson, 2012), which enables us to track and understand changes and developments (Bowen, 2009; Gross, 2018; O’Connor, 2011) in current or future healthcare needs (Atkinson & Coffey, 2010; Bowen, 2009) and in healthcare organizations or social settings (Coffey, 2014; Mercieca et al., 2019).

The research questions in document analysis can be either *pre-determined* or *exploratory*, depending on the information that is available. Pre-determined research questions are appropriate if there is previous scientific knowledge and sufficient information on the data that the contents of documents can provide (Kallio et al., 2018, 2020; Puustinen et al., 2021). Information or access may be unavailable before the data collection if highly confidential or sensitive data are involved. This means that the research questions are exploratory during this phase and can only be specified after an overview of the entire data has been obtained (Häggman-Laitila et al., 2019, 2020; Kangasniemi et al., 2022; Papinaho et al., 2021, 2022; Toivonen et al., 2020). Determining the research questions and data can be based on previous studies and by consulting responsible authorities (Appleton & Cowley, 1997) who hold relevant data or experts and practitioners engaged in the study topic. In addition, an empirical field study can be carried out to acquire sufficient knowledge to determine the research question.

It is important to identify the most relevant characteristics of the data provided by the *documents* used in any analysis. This includes evaluating whether the type (Atkinson & Coffey, 2010; Gorichanaz & Latham, 2016) or form (Appleton & Cowley, 1997; Kaae & Traulsen, 2015; Platt, 1981) of document is suitable for the study and the document’s author and/or audience. This phase includes providing reasons to justify the use of selected documents in the analysis, by identifying available and potentially suitable documents and any bias they may have (see phase 6). Justifying the use of documents is also crucial when using highly protected or sensitive data, such as complaints from clients and patients or official investigations (Bowen, 2009; Häggman-Laitila et al., 2019, 2020; Kangasniemi et al., 2022; Papinaho et al., 2021, 2022; Toivonen et al., 2020).

The study *design* in the document analysis can be cross-sectional, longitudinal, or a case report, depending the purpose of the study and the documents that are available. In addition, document analysis can be conducted with different methodological approaches (Bowen, 2009; Gross, 2018; Kaae & Traulsen, 2015; O’Connor, 2011). Qualitative methods are applicable when the purpose is to understand and describe emerging patterns, categories, or themes on the study topic (Coffey, 2014; Häggman-Laitila, 2003; Häggman-Laitila et al., 2010; Kaae & Traulsen, 2015; Miller & Alvarado, 2005). Quantitative methods can be used to describe statistics or interpret phenomenon (Häggman-Laitila et al., 2019, 2020; Hipp et al., 2020, 2021; Olson, 2012; Toivonen et al., 2020).

Document analysis can have different *focuses*, depending on the study purpose (Atkinson & Coffey, 2010; Coffey, 2014; Finnegan, 2011; Gorichanaz & Latham, 2016). First, it can be used to understand documents and their content in a specific context, such as an analysis of healthcare steering documents (Papinaho et al., 2021, 2022). For example, the analysis can focus on how the documents relate to real-life practical healthcare (Häggman-Laitila, 2003; Häggman-Laitila et al., 2010, 2019, 2020; Toivonen et al., 2020). This can include examining the philosophical aspects of documents, such as the role they play in cultural settings (Coffey, 2014; Finnegan, 2011; Gorichanaz & Latham, 2016) and what emotions they generate (Gorichanaz & Latham, 2016). Second, document analysis can focus on the intra- or intertextuality of the documents (Atkinson & Coffey, 2010; Gorichanaz & Latham, 2016), by highlighting the similarities and differences (Atkinson & Coffey, 2010; Finnegan, 2011) of the document in relation to others. Third, the analysis can focus on the social aspects of the documents, such as how they are produced, stored, and used (Atkinson & Coffey, 2010; Finnegan, 2011; Gorichanaz & Latham, 2016).

### Second Phase: Determining the Selection Strategy

The second phase of the document analysis process is to determine the document selection strategy, based on the research purpose (Gross, 2018; O’Connor, 2011). The aim of this phase is to set limits on what is analyzed so that representative and unbiased data can be produced (Figure 2).

The *time limitations* for the selection need to be set and decisions need to be made about the *sampling strategy* and whether this should be total, random, or purposeful (Miller & Alvarado, 2005). If purposeful sampling is used, then researchers have to decided how, and when, the data saturation will be identified (Finnegan, 2011; Miller & Alvarado, 2005; Scott, 2011; Siegner et al., 2018). The third decision is to make decisions about the *entity of document*, because a document can comprise several parts or attachments (Gorichanaz & Latham, 2016). For example, the entity of a steering document produced by a ministry can be only be one document (Kallio et al., 2018) but patients records (Häggman-Laitila et al., 2019, 2020; Hipp et al., 2020, 2021; Toivonen et al., 2020) or clients’ complaints may comprise several sheets (Kangasniemi et al., 2022). These sheets, or the parts of a document, can have an equal or hierarchical relation to each other. For example, when possible disciplinary cases are investigated, the file usually contains an administrative decision as well as the investigative material used to reach that decision (Papinaho et al., 2021, 2022). The fourth decision is to determine the *inclusion and exclusion criteria* for the content of the document (Bowen, 2009; O’Connor, 2011), to ensure credible responses to the research questions. The criteria can relate to the scope, form, or expressions of information in the documents.

### Third Phase: Selecting or Developing the Extraction Matrix

The aim of the third phase is to select, or develop, the extraction matrix so that this provides a credible tool for systematically recording and extracting document data (Bowen, 2009; Gibson & Brown, 2011; Hall & Rist, 1999; Kaae & Traulsen, 2015; Figure 2). The content and structure of the extraction matrix depends on the purpose of the study, previous knowledge on the topic, and the content and form of the documents that are used. The extraction matrix can include structured, semi-structured, or open-ended items that can be used to extract data for the qualitative and/or quantitative analysis of the documents. The extraction matrix can be an *existing*, previously published structure for data extraction, such as existing care classifications (Puustinen et al., 2021). In addition, the structure of certain existing documents, such as patient records, can be used as an extraction matrix.

Deductive or inductive development strategies can be used if an existing extraction matrix is not available or suitable. A *deductive development strategy* can be used to develop an extraction matrix that is based on previous knowledge, using either a systematic review method or systematic literature searches. If previous knowledge is limited, or unavailable, experts in the field can be consulted as part of the development process (Häggman-Laitila et al., 2019, 2020; Hipp et al., 2020, 2021; Papinaho et al., 2021, 2022; Toivonen et al., 2020). For example, we consulted relevant authorities when we developed an extraction matrix to study administrative decisions related to unprofessional conduct. During this phase there may be several questions about the extraction matrix and it can be used as a preliminary method of data extraction. The number of items can then be reduced during the pilot phase of the data collection (Hipp et al., 2020, 2021; Papinaho et al., 2021, 2022).

An *inductive development strategy* can be used for the extraction matrix if previous literature is not available, the purpose of study is to provide a new point of view or if the structure of the documents are the same or not known. The first step in developing an inductive extraction matrix is to understand the entire data, then develop the items using a thematic or category-based strategy (Häggman-Laitila, 2003; Häggman-Laitila et al., 2010). For example, we used an inductive development strategy for a study on clients’ complaints, because of the heterogeneity of the structure of the documents and the content of the complaints. This method can be combined with a *deductive-inductive strategy* (Kangasniemi et al., 2022).

### Fourth Phase: Pilot Testing the Selection Strategy and Extraction Matrix With Sub-Data

The fourth phase is to pilot test the selection strategy and extraction matrix, to ensure rigor and consistent data extraction. The time limitations, sampling strategy, and decisions about the entirety of the document and the inclusion and exclusion criteria can be *elaborated* at this stage and the extraction matrix can be modified. This includes removing potential overlapping or repetitive items (Kangasniemi et al., 2022). In addition, the deductive extraction matrix and item pool can be reduced, according to the document data. Pilot testing with a sub-sample of the documents has been suggested (Gross, 2018) and we have found that approximately 10% of the data is needed to *confirm* the feasibility of the extraction matrix. After the inclusion and exclusion criteria and the extraction matrix have been elaborated and modified they can be used for the entire data (Hipp et al., 2020, 2021; Kangasniemi et al., 2022; Papinaho et al., 2021, 2022).

### Fifth Phase: Collecting and Analyzing the Data

Data collection is the fifth phase of document analysis (Figure 2) and the aim of the analysis depends on the research question and chosen approach.

#### Qualitative document analysis

If qualitative methods are going to be used for document analysis, the first step is to read the entire data to get an overall understanding of it (Appleton & Cowley, 1997; Bowen, 2009). Then the analysis units, and their focus and form, can be determined. These can be a word, sentence, or entire passage of text (Bowen, 2009). The *qualitative, inductive analysis* of documents is an iterative process that combines elements from qualitative content analysis and thematic analysis. This analysis may require some level of interpretation (Atkinson & Coffey, 2010; Bowen, 2009; Caulley, 1983; Finnegan, 2011; Gross, 2018; Kaae & Traulsen, 2015; Miller & Alvarado, 2005), if the words or terms are inconsistent in the documents (Gross, 2018). The analysis aims to organize information into categories based on patterns and themes emerging from the data (Bowen, 2009; Caulley, 1983; Gross, 2018; Miller & Alvarado, 2005; Murray & Sixsmith, 2002; O’Connor, 2011; Rasmussen et al., 2012). This requires focused re-reading and reviewing of the selected documents (Bowen, 2009) with constant comparison (Bowen, 2009; Gross, 2018; Olson, 2012) to organize it so that similar themes are clustered together (Bowen, 2009). The analysis is completed, when the evidence from the documents create a consistent picture of themes. However, other qualitative methods can also be used for analyzing the data, such as grounded theory (Bowen, 2009; Gross, 2018; Murray & Sixsmith, 2002) or discourse analysis (Coffey, 2014; Gross, 2018; Murray & Sixsmith, 2002; O’Connor, 2011; Siegner et al., 2018).

When using an *extraction matrix in qualitative analysis*, the analysis units will be collected according to the matrix. After the entire data has been extracted, the items in the matrix can be reorganized, or combined in the categories and again in the categories as long as the condensation is needed (Kallio et al., 2018, 2020).

#### Quantitative document analysis

When using extraction matrix for quantitative analysis, the data can be collected (Bowen, 2009; Caulley, 1983) to enable systematic analysis. The numerical data need to be collected according to the structured items in the extraction matrix and analyzed using statistical methods (Hipp et al., 2020, 2021; Papinaho et al., 2021, 2022).

If the matrix includes structured, semi-structured, and open-ended items for verbal text collection, the expressions in the text are extracted to the matrix. After the entire data has been extracted, the text in the items need to be reduced, and condensed to variables. In addition, the items can be coded in numerical form, so that a statistical analysis can be conducted (O’Connor, 2011; Siegner et al., 2018).

### Sixth (Integrated) Phase: Ensuring Rigor of the Study

The sixth phase is the ensuring rigor of the document analysis (Figure 2). It is an integrated phase that should be carried out and reflected throughout the document analysis process. The aim is to decrease potential bias during the document selection and analysis phases.

#### Rigor of the type of the documents

*Purpose bias* needs to be assessed because healthcare documents have been produced for specific, defined purposes (Bowen, 2009; Gross, 2018; Murray & Sixsmith, 2002; Platt, 1981), and audiences (Atkinson & Coffey, 2010; Bowen, 2009; Coffey, 2014; Finnegan, 2011; Gibson & Brown, 2011; O’Connor, 2011). Bias can relate to the document’s position on an issue, whether it relates to regulations and how formal it is. In addition, the purpose of the document can influence the content, structure, and the terminology that is used. For example, healthcare documents can be based on legal requirements, but their purposes can vary because of the different roles of the organizations that produce them. They can include health plans, statements, or organizational programs that aim to steer regional, national, or international health policies (Kallio et al., 2018), patient records or plans that record whether patients’ rights have been exercised (Häggman-Laitila, 2003; Häggman-Laitila et al., 2010, 2019, 2020; Puustinen et al., 2021; Toivonen et al., 2020; Turjamaa et al., 2015), and client or patient complaints about their rights or dissatisfaction with their care (Kangasniemi et al., 2022). In addition, care orders for children (Häggman-Laitila et al., 2010, 2019, 2020; Toivonen et al., 2020) and disciplinary decisions by national regulatory authorities that restrict how healthcare professionals can practice (Papinaho et al., 2021, 2022) are based on legal requirements. The strength of legislation-based documents is that they provide structured content, within and among documents, but the purpose of the document may restrict or reduce descriptions of the content. Also, the aim of documents can be to demonstrate their activities or developmental work to funders or organizations (Kallio et al., 2018, 2020) or describe desired practices as a result of care guidelines. The purpose and consequences of documents need to be considered during the selection, analysis, and reporting phases of document analysis.

*Author bias* also needs to be considered. Healthcare documents can be written by an individual person, a team of authors, or organizations who may place a particular emphasis on certain aspects of an issue (Atkinson & Coffey, 2010; Bowen, 2009; Gibson & Brown, 2011; Gross, 2018; Murray & Sixsmith, 2002; Scott, 2011). The documents can reflect the consensus reached by authors or organizations or include contributions by a number of professionals, such as in patients’ records (Häggman-Laitila, 2003; Häggman-Laitila et al., 2010, 2019, 2020; Hipp et al., 2020, 2021; Puustinen et al., 2021; Toivonen et al., 2020; Turjamaa et al., 2015) or in different parts of a document’s entity (Papinaho et al., 2021, 2022). The author or authors can reflect official authority or professional viewpoints, as in steering documents, patient records, or annual reports. Alternatively, they can reflect the views of private individuals, such as clients or patients complaining about care or healthcare professionals responding to regulatory authorities during official investigations into their conduct. Official state documents have been regarded as more credible than private documents (Hall & Rist, 1999; Scott, 2011), because they have produced by organizations where individuals’ opinions have been minimized (Scott, 2011). Author bias must also take account of the competencies or awareness (Caulley, 1983; Miller & Alvarado, 2005; O’Connor, 2011) of individual authors and how they can vary within or between documents. For example, patient records are usually written by different healthcare professionals with varying education and sometimes the authors of documents can be difficult to establish (Atkinson & Coffey, 2010; Scott, 2011). Author bias can also result from using second-hand reports on documents instead of the original texts (Murray & Sixsmith, 2002).

*Conflict of interest bias* needs to be considered and this can relate to who funded a healthcare documents and what influence they may have had on the process (Gibson & Brown, 2011).

#### Rigor of document selection and analysis

*Selection bias* can be linked to the databases that were used to create a document or to selections made by researchers. The way that electronic or manual databases or document storage are described, catalogued, or indexed can affect the accuracy of searches (Caulley, 1983; Gross, 2018; Miller & Alvarado, 2005). Selection bias can also exist because of the limited availability of documents (Appleton & Cowley, 1997; Bowen, 2009; Gross, 2018; Miller & Alvarado, 2005; Olson, 2012; Platt, 1981; Scott, 2011), as some are archived, but others are not retained (Appleton & Cowley, 1997; Bowen, 2009; Murray & Sixsmith, 2002; Scott, 2011; Sixsmith & Murray, 2001). Researchers can cause selection bias if the inclusion or exclusion criteria for cataloguing documents in archives or databases are unclear or inconsistent (Caulley, 1983; Gross, 2018; Miller & Alvarado, 2005). In addition, selection bias can occur if researchers only select or pinpoint data that support their own models and theories (Gross, 2018; Murray & Sixsmith, 2002). Researchers need to consider whether the selected data meets the study purpose and is sufficient to provide answers to the research questions. In addition, selection bias can be reduced by consistent selection throughout the data collection process. For example, the pre-defined inclusion and exclusion criteria can be pilot tested (Hipp et al., 2020, 2021; Kangasniemi et al., 2022; Papinaho et al., 2021, 2022) and two or more researchers can work together to double-check the data that are selected (Häggman-Laitila et al., 2019, 2020; Kangasniemi et al., 2022; Papinaho et al., 2021, 2022; Toivonen et al., 2020).

*Data bias* can result from document characteristics, as they can vary in structure, length, and content and provide varying amounts, and quality of, data for the analysis. Documents can also include inaccuracies, such as faults, deceptions, or translation errors (Appleton & Cowley, 1997; Caulley, 1983; Gibson & Brown, 2011; Hipp et al., 2021; Kallio et al., 2018, 2020; Murray & Sixsmith, 2002; Scott, 2011). This means that the data in documents can be unbalanced, and provided at different levels, which can complicate the analysis. Documents can include edited or unedited text (Bowen, 2009; Gibson & Brown, 2011; Olson, 2012) or form part of a larger text series (Gibson & Brown, 2011; O’Connor, 2011). Text can also be based on underlying assumptions or hidden agendas (Appleton & Cowley, 1997; O’Connor, 2011).

*Interpretation bias* can occur if documents are studied without considering their context (Appleton & Cowley, 1997) or treated as an accurate and complete record (Bowen, 2009; Coffey, 2014). Interpretation bias can also occur if the researchers are not familiar with the expressions in the text (Caulley, 1983; Hall & Rist, 1999; Murray & Sixsmith, 2002; Platt, 1981), concepts vary or there are different political views within or between documents. Double-checking the data coding phase can decrease interpretation bias.

*Reporting bias* can result from inconsistent descriptions of the document analysis process, including determination (Siegner et al., 2018) and justification of the use of the document analysis method (O’Connor, 2011; Siegner et al., 2018). In addition, it can occur because of the way the research data are selected and described, the analysis and interpretation of the documents, and any potential biases and measures taken to address them.

### Seventh (Integrated) Phase: Method Specific Research Ethics

The seventh, integrated, phase of the document analysis process is to reflect on the method specific research ethics of the study process (Figure 2; Kaae & Traulsen, 2015). It is noteworthy that ethical consideration is an integrated phase throughout the document analysis process, starting from the planning of the study.

*Research permission* or organizational approval are often needed for the document analysis method (Caulley, 1983; Murray & Sixsmith, 2002; Sixsmith & Murray, 2001) and this has to be evaluated in relation to how public the data is, who owns the data, and how the results will be presented. For example, research permission is not usually needed for data that are published on organizations’ web pages or in public documents (Kallio et al., 2020). However, researchers need to consider whether an organization should be informed if the data in their publicly available documents will be used or presented as a case study. Research permission and ethical reviews are needed when using secure or classified documents as research data (Häggman-Laitila et al., 2019, 2020; Kangasniemi et al., 2022; Papinaho et al., 2021, 2022; Toivonen et al., 2020). The organization or the holder of the protected data, such as patient records, may specify that each individual patient needs to give their informed consent for their data to be used (Hipp et al., 2020, 2021). In addition, it can be challenging when permission is restricted to certain unseen documents that do not contain the data that are required.

*Commitments to non-disclosure agreements* are important when highly secured or classified documents are used for research data. The holder of the data may require researchers to agree to non-disclosure statements that guarantee confidentiality. In addition, special arrangements for data collection can include the use of secured computers or the requirement to collect the data on the organization’s premises under supervision (Häggman-Laitila et al., 2019, 2020; Kangasniemi et al., 2022; Papinaho et al., 2021, 2022; Toivonen et al., 2020). Thus, the ethical discussion of document analysis includes reports of potential non-disclosure agreements and how they have been implemented.

*Protecting anonymity* and privacy must also be considered during document analysis (Murray & Sixsmith, 2002; Sixsmith & Murray, 2001). These can relate to individual data and data sources, but may also relate to document producers, target audiences, organizations, and health districts and emphasize the anonymity of minority groups, such as by gender, sexual orientation, or ethnicity. Special attention needs to be paid to confidential data, which may require extra steps to protect anonymity, for example by changing identifying information. In addition, researchers also need to consider whether it is necessary to anonymize publicly available data (Kallio et al., 2018, 2020).

## Discussion

Our theory review of previous methodological literature indicated that there was no systematic description of the document analysis method for healthcare documents as written data. The seven phases of the document analysis method presented in this paper follow methodological tradition, from determining the purpose of a study to reflecting on the research ethics (Gray et al., 2016). It is noteworthy that there was very little information on the formulation of research questions, the development of extraction matrixes, and the systematic consideration of bias and ethical questions during the document analysis research process. However, these phases are crucial in relation to the credibility of the study. In addition, a rigorous extraction matrix is crucial to demonstrate how the knowledge is produced. In addition, the precisely reported development of extraction matrixes will enable them to be used in other studies. This will make it much easier to examine longitudinal or comparative findings on the same research topics.

The document analysis method also has its limitations. Healthcare documents are often produced for a specific context and influenced by national health policy and legislation (Flaumenhaft, & Ben-Assuli, 2018). They also depend on authorities or professionals having the required competencies and resources to prepare documents (Alonso et al., 2020; De Groot et al., 2019). The purpose, content, or storage of documents can also be regulated, which can hinder the rigor of document analysis (Bowen, 2009). In addition, documents are usually structured, prioritized, and interpreted at least once and they provide indirect descriptions of empirical reality. However, the transparent and systematic reporting of data and selection biases strengthen the rigor and use of the results produced by the document analysis method.

Health science researchers need to pay more attention to the document analysis method in the future. Healthcare documents increasingly provide rich data that focus on multiple target audiences and perspectives and this can deepen our understanding of different aspects of health and healthcare. This enables researchers to study topics that would be otherwise out of reach and makes longitudinal study designs easily available. However, current and future healthcare documents need to be critically analyzed to identify whether they are credible for research data. For example, developing categories that assess the reliability of documents in relation to their availability, legal status, formality, and the dependability of their authors, would help researchers to make informed selections about document data. The synthesized categorization of documents would support digital data pools in healthcare and enable comparative research to be carried out on national and international levels. In future, multi-professional collaboration with healthcare providers is needed during the planning phases of healthcare documents. This would help to identify all potential healthcare documents that could also be used for research. It would also identify how future documents could provide content that increased the information needed to evaluate the quality and effectiveness of healthcare.

In addition, using existing data from documents also supports the social and environmental sustainability of research, by minimizing disturbing healthcare professionals and patients, and decreasing the environmental burdens of data collection (Patel et al., 2020). The increasing number of documents being produced, their characteristics and the development of new research methods is rapidly changing the research context of document analysis. We expect that, in the future, artificial intelligence and data mining will be able to provide knowledge that is unreachable by traditional methods (Mehta & Pandit, 2018; Sundermann et al., 2019). In addition, increasing use and availability of big data will provide a data source for document analysis method but also challenge methodological development of document analysis in the future. However, methodological starting points needs to be rigorous and repeatable (Caulley, 1983), regardless of the data collection and analysis methods (Bowen, 2009; Siegner et al., 2018). Our rigorous process for document analysis provides a basis for studies that use documents as research data.

### Limitations

There are some strengths and limitations to consider when interpreting the study findings. The theory review method was used (Campbell et al., 2014), because there was no review method available for theoretical and methodological papers. To strengthen the reliability of this study, we have reported the search strategy, including the combination of search words and the inclusion and exclusion criteria. In addition, the search query parameters were formulated in collaboration with an information specialist, to decrease the search bias. However, as we only limited our searches to publications in English, this may have caused language bias (Martín-Rodero et al., 2018). We conducted both electronic and manual searches to decrease publication bias. We included book chapters that were available on the electronic databases or identified based on the reference lists of the selected papers. Thus, there is a risk that other suitable chapters may not have been identified. The papers were selected by three independent researchers to strengthen the quality and trustworthiness of the study but the use of screening software would have decreased the human error of selection. We also used the Preferred Reporting Items for Systematic Reviews and Meta-Analyses checklist to verify the comprehensiveness of our review (Martín-Rodero et al., 2018; Moher et al., 2009). However, the quality of the reviewed publications was not evaluated, due to the lack of specific criteria for methodological papers and book chapters.

## Conclusion

The increasing number of healthcare documents provides an important source of scientific knowledge, but the scientific use of multiple documents requires systematic and transparent methods. Previous methodological literature, have not provided a systematic description of the document analysis process and little attention has been paid to formulating research questions, developing extraction matrixes, and the systematic consideration of bias and ethics. The seven-phrase document analysis method developed by this study can be used to carry out, and evaluate, document analysis studies and it contributes to the feasibility and credibility of the method. A rigorous process for document analysis method is needed to strengthen the potential, and use, of knowledge on what healthcare documents can provide in the future.

## References

[bibr1-23333936221108706] Alonso V. Santos J. V. Pinto M. Ferreira J. Lema I. Lopes F. Freitas A. (2020). Health records as the basis of clinical coding: Is the quality adequate? A qualitative study of medical coders’ perceptions. Health Information Management Journal, 49(1), 28–37. 10.1177/1833358319826351 30744403

[bibr2-23333936221108706] Appleton J. V. Cowley S. (1997). Analysing clinical practice guidelines. A method of documentary analysis. Journal of Advanced Nursing, 25(5), 1008–1017. https://doi:10.1046/j.1365-2648.1997.19970251008.x 9147206 10.1046/j.1365-2648.1997.19970251008.x

[bibr3-23333936221108706] Atkinson P. Coffey A. (2010). Analysing documentary realities. In Silverman D. (Ed.), Qualitative research (pp. 56–76). SAGE.

[bibr4-23333936221108706] Boeije H. (2002). A purposeful approach to the constant comparative method. Quality & Quantity, 36(4), 391–409. https://doi:10.1023/A:1020909529486

[bibr5-23333936221108706] Bowen G. A. (2009). Document analysis as a qualitative research method. Qualitative Research Journal, 9(2), 27–40. https://doi:10.3316/QRJ0902027

[bibr6-23333936221108706] Campbell M. Egan M. Lorenc T. Bond L. Popham F. Fenton C. Benzeval M. (2014). Considering methodological options for reviews of theory: Illustrated by a review of theories linking income and health. Systematic Reviews, 3(1), 1–11. https://doi:10.1186/2046-4053-3-114 25312937 10.1186/2046-4053-3-114 PMC4208031

[bibr7-23333936221108706] Caulley D. (1983). Document analysis in program evaluation. Evaluation and Program Planning, 6(1), 19–29. https://doi:10.1016/0149-7189(83)90041-1

[bibr8-23333936221108706] Coffey A. (2014). Analysing documents. In Flick U. (Ed.), The SAGE handbook of qualitative data analysis (pp. 367–379). SAGE. https://doi:10.4135/9781446282243.n25

[bibr9-23333936221108706] De Groot K. Triemstra M. Paans W. Francke A. L . (2019). Quality criteria, instruments, and requirements for nursing documentation: A systematic review of systematic reviews. Journal of Advanced Nursing, 75(7), 1379–1393. 10.1111/jan.13919 30507044

[bibr10-23333936221108706] Finnegan R. (2011). Using documents. In Sapsford R. Jupp V. (Eds.), Data collection and analysis (pp. 138–152). SAGE. 10.1007/978-1-4842-4492-0_1

[bibr11-23333936221108706] Flaumenhaft Y. Ben-Assuli O. (2018). Personal health records, global policy and regulation review. Health Policy, 122(8), 815–826. 10.1016/j.healthpol.2018.05.002 29884294

[bibr12-23333936221108706] Gibson W. Brown A. (2011). Using documents in research in: Working with qualitative data. In Gibson W. Brown A. (Eds.), Working with qualitative data (pp. 65–83). SAGE.

[bibr13-23333936221108706] Gorichanaz T. Latham K. F. (2016). Document phenomenology: A framework for holistic analysis. Journal of Documentation, 72(69), 1114–1133. https://doi:10.1108/JD-01-2016-0007

[bibr14-23333936221108706] Gray J. Grove S. Sutherland S. (2016). Nursing research (8th ed.). Elsevier Saunders.

[bibr15-23333936221108706] Gross J. (2018). Document analysis. In Frey B. (Ed.), The SAGE Encyclopedia of educational research, measurement and evaluation (pp. 545–548). SAGE. https://doi:10.1002/9781118901731.iecrm0071

[bibr16-23333936221108706] Häggman-Laitila A. (2003). Early support needs of Finnish families with small children. Journal of Advanced Nursing, 41(6), 1–12. 10.1046/j.1365-2648.2003.02571.x 12622868

[bibr17-23333936221108706] Häggman-Laitila A. Salokekkilä P. Satka M. Toivonen K. Kekolahti P. Ryynänen O-P . (2019). The coping of young Finnish adults after out-of-home care and aftercare services: A document-based analysis. Children and Youth Services Review, 120, 150–157. 10.1016/j.childyouth.2019.05.009

[bibr18-23333936221108706] Häggman-Laitila A. Tanninen H. -M. Pietilä A.-M. (2010). Effectiveness of resource-enhancing family-oriented intervention. Journal of Clinical Nursing, 19(17–18), 2500–2510. 10.1111/j.1365-2702.2010.03288.x 20920078

[bibr19-23333936221108706] Häggman-Laitila A. Toivonen K. Puustelli A. Salokekkilä P. (2020). Do aftercare services take young people’s health behavior into consideration? A retrospective document analysis from Finland. Journal of Pediatric Nursing, 55, 134–140. 10.1016/j.pedn.2020.08.005 32950820

[bibr20-23333936221108706] Hall A. Rist R. (1999). Integrating multiple qualitative research methods (or avoiding the precariousness of a one-legged stool). Psychology & Marketing, 16(4), 291–304. https://doi:10.1002/(SICI)1520-6793(199907)

[bibr21-23333936221108706] Hipp K. Tiihonen E. Kuosmanen L. Kangasniemi M. (2020). As needed medication events in a forensic psychiatric hospital: A document analysis of the prevalence and reasons. Journal of Forensic Mental Health, 19(4), 329–340. 10.1080/14999013.2020.1774686

[bibr22-23333936221108706] Hipp K. Tiihonen E. Kuosmanen L. Katajisto J. Kangasniemi M. (2021). Patient participation in pro re nata medication in forensic psychiatric care: A nursing document analysis. Journal of Psychiatric and Mental Health Nursing, 28(4), 611–621. 10.1111/jpm.12706 33085793

[bibr23-23333936221108706] Kaae S. Traulsen J. M. (2015). Qualitative methods in pharmacy practice research. In Babar Z.-U.-D. (Ed.), Pharmacy practice research methods (pp. 49–68). Springer International Publishing. 10.1007/978-3-319-14672-0

[bibr24-23333936221108706] Kallio H. Pietilä A.-M. Johnson M. Kangasniemi M. (2018). Environmental responsibility in university hospitals. A qualitative study of environmental programs and the views of environmental managers. Journal of Hospital Administration, 7(5), 56–69. 10.5430/jha.v7n5p56.

[bibr25-23333936221108706] Kallio H. Voutilainen A. Viinamäki L. Kangasniemi M. (2020). In-service training to enhance the competence of health and social care professionals: A document analysis of web-based training reports. Nurse Education Today, 92, 104493. 10.1016/j.nedt.2020.104493 32673934

[bibr26-23333936221108706] Kangasniemi M. Papinaho O. Moilanen T. Leino-Kilpi H. Siipi H. Suominen S. Suhonen R. (2022). Neglecting the care of older people in residential care settings: A national document analysis of complaints reported to the Finnish supervisory authority. Health and Social Care in Community, 30, e1313–e1324. 10.1111/hsc.13538 34499408

[bibr27-23333936221108706] Martín-Rodero H. Sanz-Valero J. Galindo-Villardon P. (2018). The methodological quality of systematic reviews indexed in the MEDLINE database. A multivariate approach. Electronic Library, 36(1), 146–158. 10.1108/EL-01-2017-0002

[bibr28-23333936221108706] Mehta N. Pandit A. (2018). Concurrence of big data analytics and healthcare: A systematic review. International Journal of Medical Informatics, 114, 57–65. 10.1016/j.ijmedinf.2018.03.013 29673604

[bibr29-23333936221108706] Mercieca S. Belderbos J. S. A. van Baardwijk A. Delorme S. van Herk M. (2019). The impact of training and professional collaboration on the interobserver variation of lung cancer delineations: A multi-institutional study. Acta Oncologica, 58(2), 200–208. 10.1080/0284186X.2018.1529422 30375905

[bibr30-23333936221108706] Miller F. Alvarado K. (2005). Incorporating documents into qualitative nursing research. Journal of Nursing Scholarship, 37(4), 348–353. https://doi:10.1111/j.1547-5069.2005.00060.x 16396408 10.1111/j.1547-5069.2005.00060.x

[bibr31-23333936221108706] Moher D. Liberati A. Tetzlaff J. Altman D. G. (2009). Preferred reporting items for systematic reviews and meta-analyses: The PRISMA meta-analyses. British Medical Journal, 339(12), b2535. 10.1136/bmj.b2535 PMC2714657 19622551

[bibr32-23333936221108706] Murray C. D. Sixsmith J. (2002). Qualitative health research via the Internet: Practical and methodological issues. Health Informatics Journal, 8(1), 47–53. https://doi:10.1177/146045820200800109

[bibr33-23333936221108706] O’Connor M. (2011). Documentary analysis and policy. In Addington-Hall J. Bruera E. Higginson I. Payne S. (Eds.), Research methods in palliative care (pp. 229–245). Oxford University Press.

[bibr34-23333936221108706] Olivares Bøgeskov B. Grimshaw-Aagaard S. L. S . (2019). Essential task or meaningless burden? Nurses’ perceptions of the value of documentation. Nordic Journal of Nursing Research, 39(1), 9–19. 10.1177/2057158518773906

[bibr35-23333936221108706] Olson M. (2012). Document analysis. In Mills A. Durepos G. Wiebe E. (Eds.), The Encyclopedia of case study research (pp. 319–320). SAGE. 10.4135/9781473914230

[bibr36-23333936221108706] Papinaho O. Häggman-Laitila A. Kangasniemi M. (2021). Unprofessional conduct by registered nurses: A document analysis of disciplinary decisions in Finland. Nursing Ethics, 19(1), 131–144. 10.1177/09697330211015289 PMC8866744 34583555

[bibr37-23333936221108706] Papinaho O. Häggman-Laitila A. Pasanen M. Kangasniemi M. (2022). Disciplinary processes for nurses, from organizational supervision to outcomes: A document analysis of a regulatory authority’s decisions. Journal of Nursing Management. Advance online publication. 10.1111/jonm.13679 PMC10084253 35562646

[bibr38-23333936221108706] Patel S. P. Webster R. K. Greenberg N. Weston D. Brooks S. K. (2020). Research fatigue in COVID-19 pandemic and post-disaster research: Causes, consequences and recommendations. Disaster Prevention Management, 29(4), 445–455. https://doi:10.1108/DPM-05-2020-0164 33679011 10.1108/DPM-05-2020-0164 PMC7932124

[bibr39-23333936221108706] Platt J. (1981). Evidence and proof in documentary research: Some shared problems of documentary research. Sociological Review, 29(1), 53–67. https://doi:10.1111/j.1467-954X.1981.tb03021.x

[bibr40-23333936221108706] Puustinen J. Kangasniemi M. Turjamaa R. (2021). Are comprehensive and individually designed care and service plans for older people’s home care a vision or a reality in Finland? Health and Social Care in the Community, 29(5), e144–e152. 10.1111/hsc.13255 33326161

[bibr41-23333936221108706] Rasmussen P. Muir-Cochrane E. Henderson A. (2012). Document analysis using an aggregative and iterative process. International Journal of Evidence-Based Healthcare, 10(2), 142–145. https://doi:10.1111/j.1744-1609.2012.00262.x 22672603 10.1111/j.1744-1609.2012.00262.x

[bibr42-23333936221108706] Ritter A. Z. Bowles K. H. O’Sullivan A. L. Carthon M. B. Fairman J. A. (2018). A policy analysis of legally required supervision of nurse practitioners and other health professionals. Nursing Outlook, 66(6), 551–559. https://doi:10.1016/j.outlook.2018.05.004 30122248 10.1016/j.outlook.2018.05.004

[bibr43-23333936221108706] Scott B. J. (2011). Documents, types of. In Lewis-Beck M. Bryman A. Liao T. (Eds.), The SAGE Encyclopedia of social science research methods (pp. 282–284). SAGE.

[bibr44-23333936221108706] Siegner M. Hagerman S. Kozak R. (2018). Going deeper with documents: A systematic review of the application of extant texts in social research on forests. Forest Policy and Economics, 92, 128–135. https://doi:10.1016/j.forpol.2018.05.001

[bibr45-23333936221108706] Sixsmith J. Murray C. D. (2001). Ethical issues in the documentary data analysis of Internet posts and archives. Analysis of Internet Posts and Archives, 11(3), 423–432. https://doi:10.1177/104973201129119109 10.1177/104973201129119109 11339083

[bibr46-23333936221108706] Sundermann A. Miller J. Marsh J. Saul M. Shutt K. Pacey M. Mustapha M. Ayres A. Pasculle W. Chen J. Snyder G. Dubrawski A. Harrison L. (2019). Corrigendum: Automated data mining of the electronic health record for investigation of healthcare-associated outbreaks. Infection Control and Hospital Epidemiology, 40(5), 314–319. 10.1017/ice.2019.84 30773168 PMC8189294

[bibr47-23333936221108706] Toivonen K. Salokekkilä P. Puustelli A. Häggman-Laitila A. (2020). Somatic and mental symptoms, medical treatments and service use in aftercare: Document analysis of Finnish care leavers. Children and Youth Services Review, 114, 105079. 10.1016/j.childyouth.2020.105079

[bibr48-23333936221108706] Turjamaa R. Hartikainen S. Kangasniemi M. Pietilä A.-M. (2015). Is it time for a comprehensive approach in older home care clients’ care planning in Finland? Scandinavian Journal of Caring Sciences, 29(2), 317–324. 10.1111/scs.12165 25308748

[bibr49-23333936221108706] Walker E. McMahan R. Barnes D. Katen M. Lamas D. Sudore R. (2018). Advance care planning documentation practices and accessibility in the electronic health record: Implications for patient safety. Journal of Pain and Symptom Management, 55(2), 256–264. 10.1016/j.jpainsymman.2017.09.018 28943360 PMC5794631

